# Host protective ASP-based vaccine against the parasitic nematode *Ostertagia ostertagi* triggers NK cell activation and mixed IgG1-IgG2 response

**DOI:** 10.1038/srep29496

**Published:** 2016-07-11

**Authors:** Ana González-Hernández, Stefanie Van Coppernolle, Jimmy Borloo, Frederik Van Meulder, Oonagh Paerewijck, Iris Peelaers, Georges Leclercq, Edwin Claerebout, Peter Geldhof

**Affiliations:** 1 Laboratory of Parasitology, Department of Virology, Parasitology and Immunology, Faculty of Veterinary Sciences, Ghent University, Belgium; 2 Laboratory of Experimental Immunology, Department of Clinical Chemistry, Microbiology and Immunology, Faculty of Medicine and Health Sciences, Ghent University, Belgium

## Abstract

The mucus-dwelling parasite *Ostertagia ostertagi* is one of the most important gastrointestinal nematodes in cattle. Our group has previously demonstrated the protective capacity of a vaccine against this parasite based on a native activation-associated secreted protein ASP1 (nASP) in combination with the saponin adjuvant QuilA. The aim of the current study was to analyse the effect of both antigen and adjuvant on the cellular and humoral vaccine-induced immune responses by comparing the native ASP to a recombinant version expressed in *Pichia pastoris* (pASP) and replacing QuilA by Al(OH)_3_. Immunization of cattle with the protective nASP+QuilA vaccine was associated with antigen-induced proliferation of natural killer (NK) cells combined with IFN-γ secretion and the induction of a mixed IgG1/IgG2 antibody response. ASP-specific activation and proliferation of NK cells was also observed in mice following the same vaccination regime. Replacing QuilA by Al(OH)_3_ or nASP by pASP significantly decreased the capacity of the vaccines to trigger both NK cell activation and antibody responses and failed to induce protection against a challenge infection. Reduction of the structurally anchoring disulphide bonds of the nASP completely abolished its ability to induce NK cell activation and antibody responses, highlighting the importance of protein conformation for the immunostimulatory activity.

Helminth infections pose a massive burden on human and animal health worldwide. Despite the widespread development of drug resistant worms, anthelmintic treatment still remains the main method to control these infections^1^,^2^. Vaccination strategies, either targeting the reduction in adult worm numbers present in the host or the reduction of worm fecundity, offer a promising alternative for anthelmintic treatment^3^,^4^. Nevertheless, hitherto only few vaccines against this type of pathogens are available. Two of the commercially available vaccines target the cattle and sheep lungworms *Dictyocaulus viviparus*^5^,^6^ and *Dictyocaulus filaria*^7^, respectively, and are based on whole irradiated larvae of these worms. Recently a promising vaccine against the blood feeding nematode *Haemonchus contortus* in sheep, based on native antigens isolated from adult worms, was commercialized^8^. However, these examples of vaccines are exceptions. Due to the complex life cycle of helminths, there are many practical issues and high costs involved in the production of high quantities of these vaccines. Therefore, mimicking the protective response by recombinant antigens would provide a major breakthrough in parasite vaccine development. Although this approach has already proven successful for the production of protective vaccines against the cestodes *Taenia saginata* and *Echinococcus granulosus*^9^, and the nematode *Teladorsagia circumcincta*^10^, it has been unsuccessful in many other cases^11^.

In recent years, our research group has developed an experimental vaccine against the mucus dwelling abomasal nematode *Ostertagia ostertagi* in cattle^12^,^13^,^14^,^15^,^16^,^17^,^18^, which is based on activation-associated secreted proteins (ASP). Intramuscular immunization of cattle with the native ASP (nASP) in combination with QuilA adjuvant raises an effective immune response, resulting in a significant reduction in faecal worm egg shedding of 56–74% during a two-month period^17^. A reduction in worm fecundity is typically the first manifestation of immunity against this parasite. Such decrease can significantly affect pasture infection levels and prevent parasitic gastroenteritis. A similar protective response is however not observed when the native antigen is replaced by a recombinant version produced in insect cells^14^. Furthermore, replacing the QuilA adjuvant by Al(OH)_3_ has also shown to completely abolish the protective effect of the native antigen^16^, indicating that both the antigen and the adjuvant are essential to achieve protection. Understanding how immunity in animals, vaccinated with the nASP-QuilA vaccine, is orchestrated might help to identify the essential features that are needed to induce protection, information which is crucial to direct future recombinant expression work. Previous research has shown that potential effector mechanisms involved in the vaccine-induced protection are antigen-specific IgG1 and IgG2 antibodies in the abomasal mucosa and increased levels of granule exocytosis, involving the local release of granulysin and granzyme B^18^. Information on the upstream mechanisms triggered by the vaccine and how these are influenced by antigen and adjuvant is still missing. Therefore, the overall aim of the present study was to analyse and compare the effect of both antigen (native vs recombinant) and adjuvant (QuilA vs Al(OH)_3_) on the cellular and humoral vaccine-induced immune responses.

## Results

### Vaccination with nASP+QuilA, but not pASP+QuilA or nASP+Al(OH)_3_, reduces worm egg production while increasing IgG1 and IgG2 antibody levels

Animals vaccinated in study 1 with the nASP+QuilA vaccine showed a significant reduction of 59% in cumulative egg output compared to the control vaccinated group. This confirms our previous findings^17^. In contrast, no reduction of faecal egg counts (FEC) was observed following vaccination with pASP+QuilA (Supplemental Fig. 1A). Similar to the observations made in study 1, animals from study 2 vaccinated with nASP+QuilA vaccine showed a reduction of 42% in cumulative FEC compared with the control vaccinated group, whereas no reduction of FEC was observed in the pASP+QuilA and nASP+Al(OH)_3_ vaccinated groups (Supplemental Fig. 1B). For both studies, vaccination had no effect on worm counts (data not shown).

**Figure 1:**
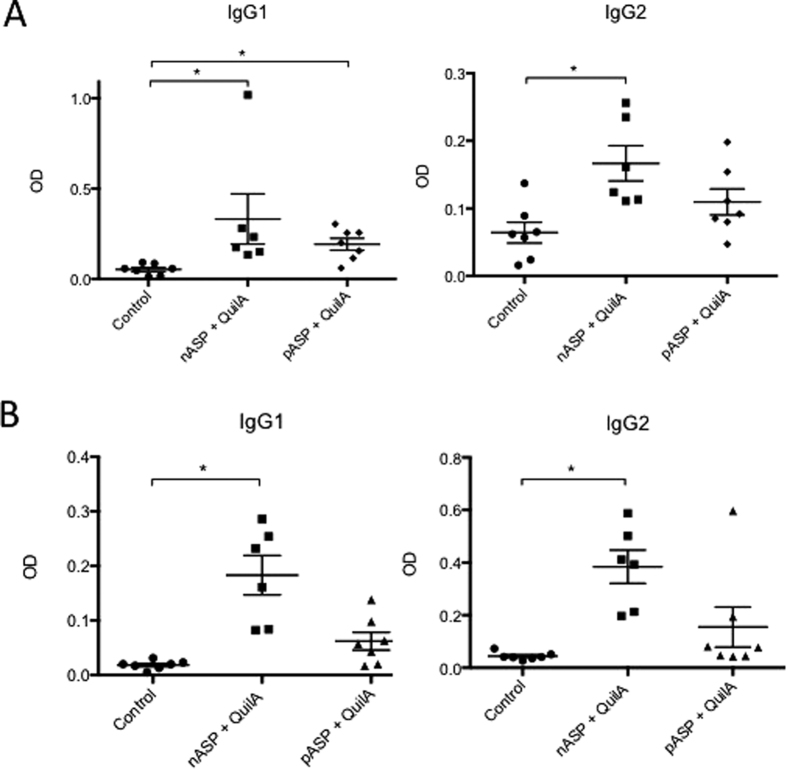
Detection of nASP-specific IgG1 and IgG2 antibodies in blood and mucosa after vaccination of cattle with nASP+QuilA and pASP+QuilA. In cattle study 1, (**A**) serum and (**B**) abomasal samples were collected from all animals and used for the detection of nASP specific IgG1 and IgG2 type antibodies through ELISA. The graphs show the individual and mean (± SEM) OD’s for each individual animal within each group ± SEM. Statistically significant differences are indicated with *p < 0.05.

Vaccination with the nASP+QuilA vaccine in study 1 resulted in a significant increase of nASP-specific IgG1 and IgG2 levels in both serum and abomasal mucus samples compared to QuilA control animals (Fig. 1A,B). Vaccination with the pASP+QuilA vaccine resulted in a significant increase of nASP-specific IgG1 levels in serum, whereas no significant changes were observed for cross-reactive systemic IgG2 and mucosal IgG1 and IgG2 levels (Fig. 1A,B). For study 2, nASP+QuilA vaccinated animals had increased levels of nASP**-specific IgG1 in both serum and mucosa, whereas this effect was less pronounced for the pASP+QuilA group and completely absent in the nASP+Al(OH)_3_ group (Supplemental Fig. 2A,B). In contrast to the first study, vaccination had a smaller impact on the amount of nASP-specific IgG2 antibodies, both systemically and in the mucosa.

Vaccine-induced IgA’s were not detectable in the serum and there were no significant increases in mucosal nASP-specific IgA levels in both studies (data not shown).

### Antibodies raised by the native and the recombinant vaccines differ in their binding properties

**Figure 2:**
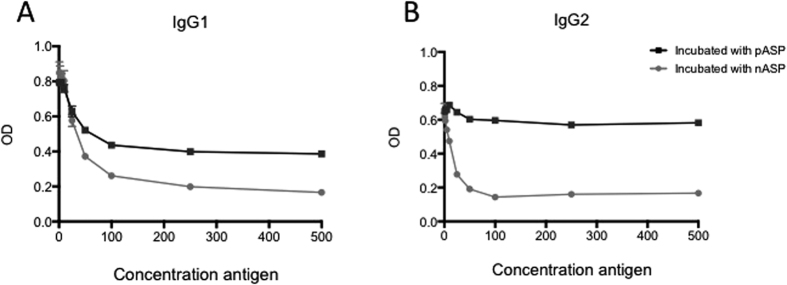
Comparison of the interaction between antibodies raised against nASP with the native and recombinant ASP. (**A**) IgG1 and (**B**) IgG2 antibodies from calves vaccinated with nASP+QuilA were evaluated in an inhibition ELISA. Graphs were generated from pooled samples in duplicate, where each point indicates the mean OD.

To evaluate the specificity of the antibodies raised by the nASP+QuilA vaccine, an inhibition ELISA was performed with serum samples collected one week after the last vaccination from nASP+QuilA vaccinated animals. As shown in Fig. 2A, pre-incubation of the serum with pASP failed to completely inhibit the binding of the IgG1 and IgG2 antibodies to nASP. In contrast, pre-incubation with nASP almost completely inhibited IgG1 and IgG2 binding to nASP.

### Vaccination of cattle with nASP+QuilA induces memory-like NK cells

**Figure 3:**
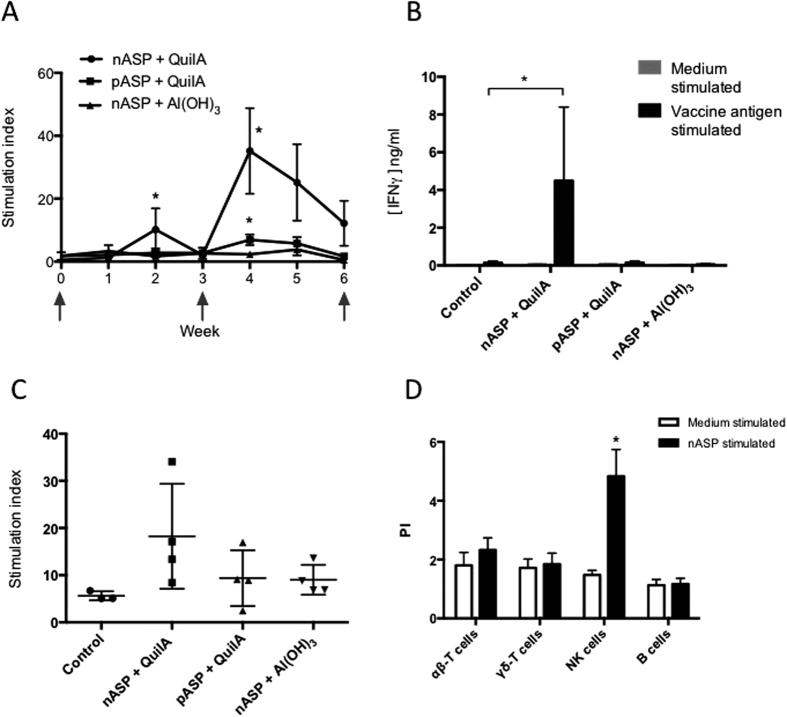
Vaccination with nASP+QuilA triggers systemic nASP-specific IFNγ producing cells and mucosal NK cells. (**A**) During cattle study 2, peripheral blood mononuclear cells (PBMCs) were isolated weekly during the vaccination period and re-stimulated with either medium alone or the antigen used in the vaccine formulation. The arrows indicate the time points at which the animals were vaccinated. ^3^H-thymidine (^3^HT) uptake was used as a measure of proliferation. The graph shows the mean stimulation index ± SEM for all vaccinated groups. (**B**) Five weeks after the first vaccination, PBMCs from nASP+QuilA, pASP+QuilA and nASP+Al(OH)_3_ groups were stimulated for 5 days with 5 μg/ml nASP or pASP. IFNγ production of these cells was determined in the supernatants using an ELISA. The graph shows the mean concentration of IFNγ for each group ± SEM. (**C**) At time of necropsy, 5 weeks following the last vaccination, abomasal mononuclear cells (MNCs) were isolated, re-stimulated, and their proliferative capacity was measured as for the PBMCs. Mean stimulaton index ± SEM is shown for all groups. (**D**) Additional assays with PKH were performed on abomasal lumph nodes (LNs) to identify the proliferative populations of the nASP+QuilA vaccinated group. After flow cytometric analysis, the proliferation results were calculated and presented as proliferation index. Statistically significant differences for all graphs are indicated with *p < 0.05.

In cattle study 2, peripheral blood mononuclear cells (PBMCs) were isolated on a weekly basis and used for phenotypical and functional analysis to measure antigen-specific proliferation *in vitro*. No vaccination-induced changes were observed in frequencies of αβ-T cells, γδ-T cells, B cells or NK cells in the PBMC fraction (Supplemental Fig. 3A–D). However, when PBMCs were re-stimulated *in vitro* with the vaccine antigens, nASP for the nASP+QuilA and nASP+Al(OH)_3_ vaccinated animals and pASP for the pASP+QuilA, antigen-specific proliferation was mainly found in the nASP+QuilA group (Fig. 3A). Proliferation was the highest in the nASP+QuilA vaccinated animals and became significantly different from control animals on weeks 2 and 4 after the first vaccination, with a decline thereafter (Fig. 3A). In the pASP+QuilA vaccinated group, antigen-specific proliferation only became significantly different from control animals one week after the first booster vaccination (Fig. 3A), but also here, a decline in proliferation was observed afterwards. Proliferation in the nASP+Al(OH)_3_ vaccinated group (Fig. 3A) and in control animals (data not shown) was not significantly different throughout the whole vaccination period.

In order to determine whether *in vitro* re-stimulated PBMCs produced specific cytokines linked to either a Th1 or a Th2 type immune response, IFNγ levels were measured in culture supernatants produced by PBMCs isolated at week 5 and stimulated either with medium alone or with 5 μg/ml of the vaccine antigen for five days. IFNγ levels were significantly higher in the culture supernatant of the cells from the nASP+QuilA group, whereas no effect was observed for the other groups (Fig. 3B). There was no detectable IL-4 production after *in vitro* culture of PBMCs isolated from any group of animals with medium nor with the vaccine antigens (data not shown).

Next, to characterize the cellular immune response in the abomasum following vaccination and infection, abomasal lymph nodes (LNs) from all animals were obtained at the time of necropsy (i.e. five weeks after the onset of the trickle infection) for phenotypical analysis and proliferation assays. No statistical differences were found in the frequencies of αβ-T cells, γδ-T cells, B cells or NK cells in any vaccine protocol (Supplemental Fig. 4A–D). Similar to the observations made with PBMCs, there was a trend in higher proliferation following re-exposure *in vitro* in the group of animals that was vaccinated with the protective nASP+QuilA vaccine (Fig. 3C), although this did not reach statistical significance. These responding cells were subsequently characterized by isolating and labelling the abomasal LN cells of nASP+QuilA vaccinated animals with PKH prior to *in vitro* re-stimulation with the nASP. As shown in Fig. 3D, proliferation was mainly detected in the CD335^+^ cell population, where CD335 expression is typically used to define NK cells.

Finally, immunohistofluorescence and immunohistochemical stainings were conducted on tissue sections from the abomasum. No significant differences in the numbers of T cells, B cells, macrophages, mast cells nor globular leukocytes were observed between the vaccinated groups and the control group (Supplemental Fig. 5A–E). In addition, intraepithelial and lamina propria lymphocytes were isolated from the abomasum of all animals at the time of necropsy to determine the frequencies of αβ-T cells, γδ-T cells, B cells and CD335^+^ cells. No differences were observed in the frequencies of these cells between the different vaccinated groups (Supplemental Fig. 6A–D).

### Vaccine-induced immune responses in a murine immunization model are similar to those observed in cattle

**Figure 4:**
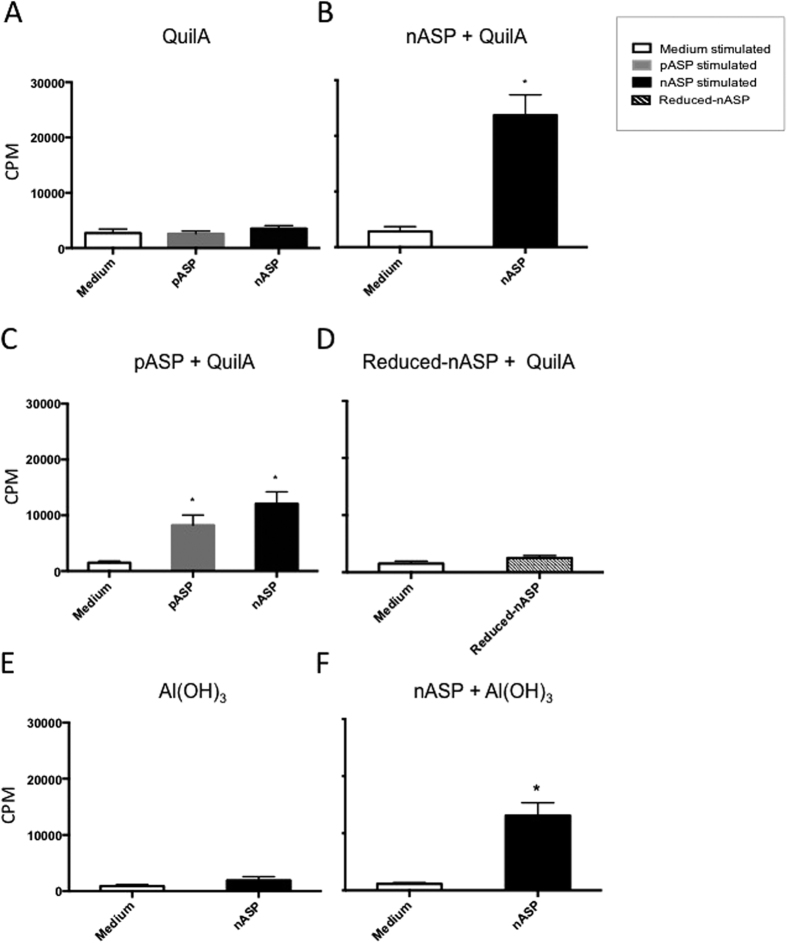
Vaccination of mice with nASP+QuilA induces higher nASP-specific cell proliferation than other vaccine formulations. Mice were immunized three times in a 3-week interval intramuscularly in the thigh muscle and euthanized one week following the last vaccination. Mouse spleen MNCs were isolated and re-stimulated with either medium alone, nASP or pASP. ^3^HT uptake was used as a measure of total cell proliferation. All graphs show the mean counts per minute (CPM) ± SEM for animals vaccinated with (**A**) QuilA, (**B**) nASP+QuilA, (**C**) pASP+QuilA, (**D**) Reduced-ASP+QuilA, (**E**) Al(OH)_3_ and (**F**) nASP+Al(OH)_3_. Statistically significant differences are indicated with *p < 0.05.

Based on the results obtained in cattle, we subsequently investigated whether the observed NK cell response following vaccination with the nASP+QuilA vaccine was unique to cattle or whether this would also be triggered in mice. To characterize the cellular immune response following vaccination in mice, spleen mononuclear cells (MNCs) were isolated at time of necropsy, and used for both ^3^H-thymidine (^3^HT) and PKH proliferation assays to determine antigen-specific proliferation *in vitro*. As observed in cattle, cells isolated from mice solely vaccinated with either QuilA or Al(OH)_3_ did not show any proliferation following *in vitro* stimulation with the nASP or pASP (Fig. 4A,E). Although significant proliferation was observed in cells from the pASP+QuilA and nASP+Al(OH)_3_ vaccinated animals following re-stimulation with the corresponding vaccine antigens (Fig. 4B,C,F), the highest proliferation was again observed in the nASP+QuilA group. Furthermore, exposure of the cells from the pASP+QuilA animals to nASP also resulted in a significant cellular proliferation in comparison to the medium control (Fig. 4C). Interestingly, cells from mice vaccinated with the nASP of which the disulphide bonds were reduced did not show any proliferation after re-stimulation with the reduced antigen (Fig. 4D).

**Figure 5:**
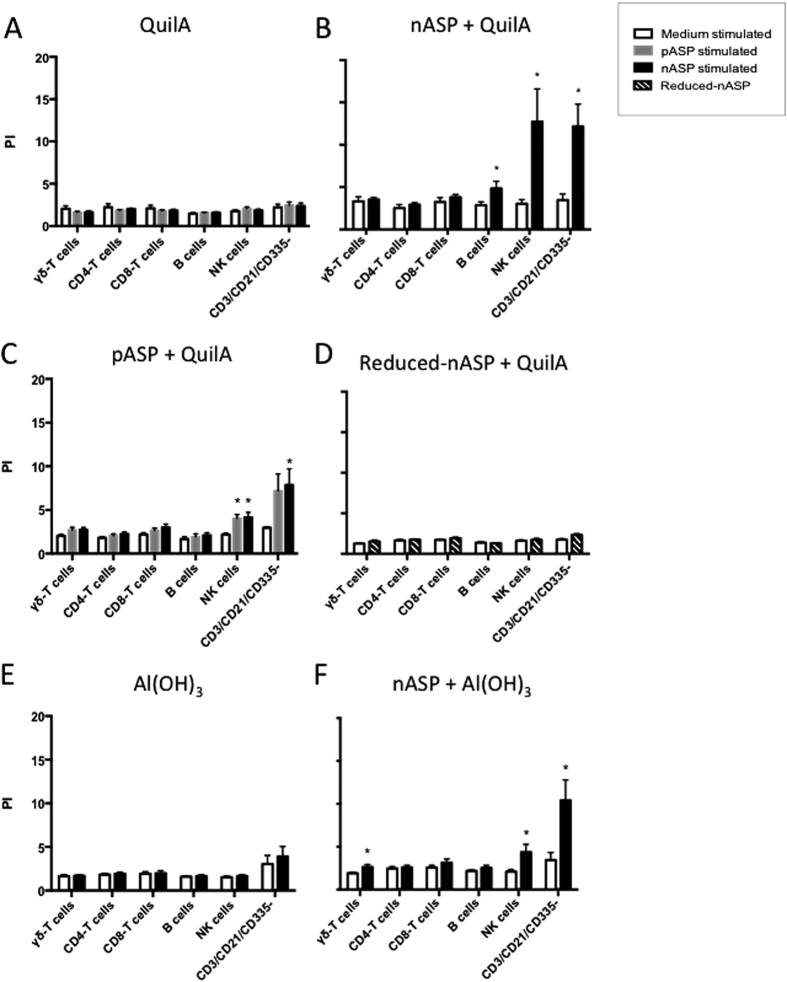
NK cells and CD3^−^/CD21^−^/CD335^−^ cells are the main vaccine-induced proliferative cell populations in mice. Mouse spleen MNCs were isolated and re-stimulated for 5 days with either medium alone, nASP or pASP and stained with monoclonal antibodies for further FACS analysis. PKH incorporation to the membrane and its further dilution was used as a measure of cell proliferation. All graphs show the mean proliferation index (PI) ± SEM for animals vaccinated with (**A**) QuilA, (**B**) nASP+QuilA, (**C**) pASP+QuilA, (**D**) Reduced-ASP+QuilA, (**E**) Al(OH)_3_ and (**F**) nASP+Al(OH)_3_. Statistical differences are indicated with *p < 0.05.

PKH assays were subsequently performed in order to characterize the proliferative cell populations. Confirming the ^3^HT assays (Fig. 4), Fig. 5 shows that there was no increased proliferation of cells from the QuilA and Al(OH)_3_ control groups nor from the reduced-nASP+QuilA group upon *in vitro* stimulation with nASP, pASP or reduced-nASP (Fig. 5A,D,E). On the other hand, B cells, and especially NK and non-T, non-B non-NK cells (CD3^−^/CD21^−^/CD335^−^) from nASP+QuilA vaccinated mice showed significant proliferation after *in vitro* re-stimulation with nASP compared to cells stimulated with medium alone (Fig. 5B). Antigen-specific proliferation of the NK and non-T, non-B non-NK cells (CD3^−^/CD21^−^/CD335^−^) following re-stimulation with nASP was also observed with cells from the pASP+QuilA and nASP+Al(OH)_3_ groups (Fig. 5C,F), albeit at a lower level compared to the nASP+QuilA group. When re-stimulating cells from the nASP+Al(OH)_3_ group with nASP, modest levels of γδ-T cell proliferation could also be observed (Fig. 5F). Finally, re-stimulation of pASP+QuilA cells with pASP induced antigen-specific NK cells proliferation, which was similar to that observed after nASP stimulation, but again much lower than the levels observed in the nASP+QuilA group (Fig. 5C).

**Figure 6:**
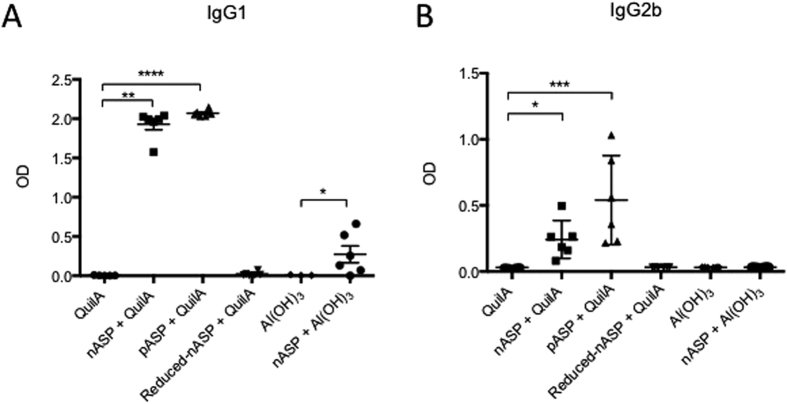
Detection of nASP-specific IgG1 and IgG2b antibodies in blood after vaccination of mice with different vaccine formulations. Mice serum was collected at time of necropsy and used for the detection of nASP specific (**A**) IgG1 and (**B**) IgG2b type antibodies through ELISA analysis. The graph shows the OD for each individual animal within each group ± SEM. Statistical differences are indicated with *p < 0.05, **p < 0.005, ***p < 0.001, ****p < 0.001.

At the time of necropsy, all mice were bled from the retro-orbital vein and the serum was used to measure antigen specific IgG1, IgG2a and IgG2b levels by enzyme-linked imminosorbent assay (ELISA), essentially as described for cattle. The levels of nASP-specific IgG1 antibodies were significantly higher in animals vaccinated with nASP+QuilA, pASP+QuilA and nASP+Al(OH)_3_ (Fig. 6A) compared to their respective controls. No significant increase in antigen-specific IgG1 levels could be detected in the reduced-nASP+QuilA and Al(OH)_3_ immunized animals. While no significant levels of nASP-specific IgG2a could be detected in any of the groups (data not shown), IgG2b antibodies were significantly higher in animals vaccinated with nASP+QuilA and pASP+QuilA when compared to the QuilA controls, whereas no significant increase could be observed for the other groups (Fig. 6B).

## Discussion

NK cells were the major cell population found in nASP+QuilA vaccinated animals which proliferated following re-stimulation of MNCs from the abomasal draining LN or spleen of cattle and mice, respectively. NK cells are large granular lymphocytes that either become directly activated by pathogens or through interaction with other pathogen-activated immune cells. They were originally named after their ability to spontaneously kill transformed, infected or non-self cells without prior sensitization or activation. Despite their innate origin, there is accumulating evidence that also NK cells can develop long-lived and highly specific memory to a variety of antigens^19^,^20^,^21^. Previous studies also indicate that NK cells are likely to be involved in the natural immune response in cattle following nematode infection. It was already shown that NK cells are important in the proliferation observed in the abomasal lymph nodes during the course of an infection^22^,^23^. More recently, an antigen-specific proliferation of NK cells was observed in the draining lymph nodes of the small intestine of animals infected with *C. oncophora*^24^. Also, analysis of transcriptional changes by qRT-PCR in the abomasal mucosa following *Ostertagia* infection showed a significant upregulation of the NK cell marker CD335^25^ and the expression of granulysin and granzyme, both proteins that are typically produced by NK cells^18^. Furthermore, the flow cytometric analyses performed in this study showed that NK cells represented roughly 15% of the total abomasal lymphocyte population following infection.

The observed NK cell activation and IFNγ production following *in vitro* re-stimulation with the native ASP is also in line with previously published data on an ASP (rOv-ASP-1) from the filarial parasite *Onchocerca volvulus.* In a series of papers, it was shown that immunisation with Ov-ASP-1 induced a highly dominant IFN-γ recall response following vaccination, most likely produced by activated NK cells, combined with a mixed IgG1/IgG2 antibody reaction^26^,^27^,^28^. Similar to the *O. ostertagi* ASP, the bioactivity of Ov-ASP-1 was completely lost after denaturing the protein by boiling^26^. It is also interesting to note that secreted protein(s) of the human hookworm *Necator americanus* selectively bind to NK cells and induced IFN-γ production and that this process is dependent on the presence of the cytokines IL-2 and IL-12, likely secreted by activated antigen-presenting cells^29^,^30^. Whether ASPs are responsible for this activity is currently unknown.

Teixeira-Carvalho *et al*.^30^ previously hypothesized that the activation of NK cells and IFN-γ production is part of an immune evasion strategy by the worms, in an attempt to down-regulate a protective Th2 response^29^. In case of the *O. ostertagi* vaccine it is unclear whether the NK cell activation is necessary to obtain protection or, alternatively, whether the protective capacity of the vaccine is based on the generation of antibodies, potentially interfering with the immunomodulatory activity of the ASP. Over the years, several studies have demonstrated that antibodies can provide protective immunity against gastrointestinal helminths in a direct and/or indirect way^31^. This protection would hypothetically be achieved through antibody-dependent activation of immune cells in the intestinal mucosa, or by interfering with enzymes and processes required for larval migration, invasion and feeding. In that context, nASP+QuilA vaccinated cattle had an increased amount of nASP-specific IgG1 and IgG2-type antibodies both in blood and in the abomasal mucus. Interestingly, NK cells express FcγRIII (CD16) at their surface, which can mediate antibody-dependent cytotoxicity through the recognition of sequestered antigens^32^. NK cells are highly abundant in the intraepithelial and lamina propria lymphocyte fractions of the abomasum, creating a high chance for effective contact between their CD335^+^ receptor and the different parasitic life stages of *O. ostertagi*. Therefore, a possible mechanism of action for NK cells against *O. ostertagi* could be the recognition of antibody-sequestered nASP and subsequent antibody mediated degranulation. Since NK cells are known to express granzyme B, perforin and granulysin, this could be an interesting link with previous observations on the upregulation of granulysin and granzyme B in animals protected by vaccination against *O. ostertagi*^18^.

The cellular and humoral responses observed for the protective nASP+QuilA vaccine were markedly higher compared to responses observed for the non-protective nASP+Al(OH)_3_ vaccine, both in cattle and mice. The exact mechanisms by which both adjuvants act are not completely understood yet. It has been shown that Al(OH)_3_ exerts a reservoir function, from which antigen is released slowly for a prolonged period of time^33^, whereas saponin-based adjuvants such as QuilA exert a more cytolytic function^34^. Despite the fact that Al(OH)_3_ is regarded as a Th2 type immune response inducing adjuvant, saponin-based adjuvants seem to induce a stronger antibody response against the vaccine antigen than Al(OH)_3_^35^, supporting the hypothesis of a possible important role for antibodies in the vaccination-induced protection against *O. ostertagi*. Additionally, not only the choice of adjuvant, but also the conformation of the antigen has proven to be essential in triggering a protective immune response. Unfolding the ASP completely abolished its ability to induce both NK cell activation and antibody induction. Furthermore, we also showed that the recombinant version of the ASP, expressed in *P. pastoris*, was less potent in triggering both a cellular and humoral response. These observations indicate that protein folding, potentially in combination with N- and/or O-glycans present on the peptide core, of the native ASP is crucial for its immunoreactivity and that these factors could be at the basis of the inability of recombinant ASP in triggering a similar response. This is in line with the results of inhibition ELISA’s showing that antibodies raised upon nASP vaccination preferentially bind nASP over pASP, suggesting the presence of either different and/or additional epitopes.

In conclusion, the outcome of this study indicates that immunization of animals with the protective nASP+QuilA vaccine is associated with antigen-induced proliferation of NK cells and the production of antigen-specific IgG1 and IgG2 antibodies. Replacing either: i) the native antigen by a recombinantly produced version or, ii) the QuilA adjuvant by Al(OH)_3_ both had a significant impact on the cellular and humoral vaccine-induced responses. Whether NK cells and antibodies are actually essential for conferring protection against an *O. ostertagi* challenge infection remains unknown. Also, the molecular and cellular mechanisms underlying the NK cell activation and the structural elements of the nASP that are crucial in this process require further research.

## Materials and Methods

### Native and recombinant antigen production

For the production of nASP, helminth‐naive calves were infected with 200,000 *O. ostertagi* infective larvae (L3) and euthanized 3 weeks later to collect adult worms from the abomasum. Adult worms were cultured, and excretory-secretory proteins collected from the culture supernatant as previously described^17^. The ASP sub‐fraction was purified from total adult ES material by thiol-sepharose chromatography, followed by anion exchange chromatography^17^. The protein profile of the obtained material was checked by separation on a 10% SDS-PAGE gel under reducing/denaturing conditions and visualized by Coomassie blue staining. Reduction of nASP was achieved by incubating nASP with 7.5 mM dithiothreitol (DTT) for 15 minutes at 60 °C. Afterwards, the nASP was incubated in iodo acetamide (IAA) at 37 °C for an additional 30 minutes followed by a dialysis against 150 mM phosphate-buffered saline (PBS).

Recombinant ASP was produced in *Pichia pastoris* as previously described^36^ and will be referred to as pASP.

### Immunization experiments in cattle

All animal experiments were conducted in accordance with the E.U. Animal Welfare Directives and VICH Guidelines for Good Clinical Practice, and ethical approval to conduct the studies were obtained from the Ethical Committee of the Faculty of Veterinary Medicine, Ghent University (EC2009/105, EC 2011/183). Two vaccination studies were carried out in cattle, essentially as previously described^13^,^16^. The aim of study 1 was to analyse and compare the humoral responses induced by the native and recombinant antigens in combination with QuilA. Twenty-one male crossbreed Holstein calves (6 to 8 months of age) were randomly divided over three groups of 7 animals (QuilA control, pASP+QuilA and nASP+QuilA). A second study was performed to analyse and compare the cellular responses induced by the different versions of the vaccine, i.e. nASP+QuilA, pASP+QuilA, nASP+Al(OH)_3_, in comparison to control animals vaccinated with PBS. For this study, a Holstein crossbreed population of 16 male helminth-free calves (6 to 8 months of age) was randomly divided into four groups of four animals (PBS control, nASP+QuilA, pASP+QuilA and nASP+Al(OH)_3_). For both studies, all animals were immunized three times intramuscularly in the neck with a 3-week interval. Control animals received either 750 μg of QuilA (study 1) or 1 ml of PBS (study 2) per immunization, while the animals of the nASP+QuilA, pASP+QuilA and nASP+Al(OH)_3_ groups received 30 μg of antigen in combination with either 750 μg of QuilA or an equal volume of Al(OH)_3_ adjuvants (both from Superfos Biosector, Denmark) per immunization. All animals were challenged with a trickle infection of 25,000 L3 larvae (1000 L3/day; 5 days/week, during 5 weeks), which started at the day of the third immunization. Calves from study 1 were euthanized 3 weeks after the last infection, while the animals from study 2 were euthanized immediately after the last infection. Parasitological parameters (i.e. FEC and worm counts) were analysed as described in previous trials^13^,^16^. Additionally, in study 2, abomasal LNs, abomasal epithelium and lamina propria were also isolated at time of necropsy.

### Immunization experiments in mice

All animal experiments were conducted in accordance with the E.U. Animal Welfare Directives and VICH Guidelines for Good Clinical Practice, and ethical approval to conduct the studies were obtained from the Ethical Committee of the Faculty of Veterinary Medicine, Ghent University (EC 2014/33 and EC 2014/104). Thirty-six 8-week-old C57BL/6N mice (Harlan laboratories) were divided into 6 groups of 6 animals each and immunized three times in a 3-week interval intramuscularly in the thigh muscle. Control groups received 20 μg of QuilA or Al(OH)_3_ (same volume as used in the antigen-vaccinated group) per immunization. Groups that included antigen in the vaccine formulation received either 5 μg of nASP, pASP or reduced-nASP in combination with 20 μg of QuilA adjuvant, while the last group received 5 μg of nASP in combination with an equal volume of Al(OH)_3_ per immunization. At time of necropsy (one week after the third immunization), spleens and blood from the retro-orbital vein were collected for cellular and humoral analyses.

### Isolation of mononuclear cells

For cattle, PBMCs were isolated weekly from blood by Lymphoprep (Nycomed Pharma) gradient centrifugation. Lymph node MNCs were isolated from the draining LNs of the abomasum by homogenization through mechanical disruption of the tissue followed by a Lymphoprep gradient centrifugation. Mucosal MNCs were isolated by removing the mucus from the abomasum and separating the mucosa from the submucosa. The mucosa was then cut into pieces of approximately 1 cm and extensively washed in Calcium and Magnesium free (CMF) HBSS (Invitrogen) containing 2 mM DTT (Biosolve). The DTT was removed by washing the tissue with CMF HBSS, after which the tissue was incubated for 30 minutes at 37 °C in CMF HBSS containing 5mM EDTA (Invitrogen), while gently stirring. The cells in the supernatant were collected and washed with CMF HBSS. The remaining tissue was washed with CMF HBSS and afterwards minced into small pieces of approximately 2 mm and digested with 2 mg/ml collagenase (Invitrogen) for 1 hour at 37 °C, while gently stirring. The cells in the supernatant were collected and washed with CMF HBSS and added to the cell pellet that was obtained earlier (see above). All cells were resuspended in 40% Percoll (GE Healthcare) and layered over a 62% Percoll layer. After centrifugation, all mononuclear cell fractions were isolated, washed and counted prior to cell culture or flow cytometric analysis.

To isolate MNCs from mice, spleens were removed, mechanically disrupted and homogenized, and passed through a 70 μm cell strainer (BD Biosciences). Erythrocytes were lysed with ACK lysing buffer (Invitrogen) and remaining cells were washed and counted prior to cell culture or flow cytometry analysis.

### Antibodies and Flow Cytometry

Cells were labelled in PBS containing 1% Bovine Serum Albumin (BSA), 0.1% Na-azide (both from Sigma-Aldrich) and the antibodies at the concentration recommended by the supplier. After an initial incubation of 20 minutes, the cells were washed and, whenever necessary, subsequently stained with fluorescently labelled secondary antibodies at the concentration recommended by the supplier. The cells were then incubated for 20 minutes prior to washing and resuspension in PBS, and immediately analysed using either FACS Canto or FACS Aria III flow cytometers (BD Biosciences). Non-viable cells were excluded of the analysis based on their propidium iodide (Molecular Probes) uptake.

Primary antibodies used in cattle were: non-labeled CD3 (MM1A, IgG1), TCRγδ (GB21A, IgG2b), CD21 (BAQ15A, IgM) (all from VMRD) and Alexa Fluor 488-labeled CD335 (AKS1, IgG1, AbD Serotec). Secondary antibodies used were: goat anti-mouse IgG1-FITC (Santa Cruz Biotechnology), goat anti-mouse IgG2a-APC (Invitrogen), rat anti-mouse IgG1-APC (BD Biosciences), rat anti-mouse IgG2b-FITC (Southern Biotech), rat anti-mouse IgM-APCCy7 (Biolegend) and goat anti-mouse IgG2a- PE (Invitrogen).

Antibodies used for mice were: CD3-PECy7 (clone 145-2C11), CD4-Biotin (clone RM4-5) and CD8a-PECy7 (clone 53-6.7) from eBioscience, and CD3-APC (clone 145-2C11), CD335-AlexaFluor647 (clone 29A1.4), CD19-Biotin (clone 1D3) and TCRγδ-FITC (clone GL3) from BD Biosciences. Biotin-conjugated antibodies were fluorescently labelled using streptavidin-APC-eFluor780 (eBioscience).

### Proliferation assays

The MNCs collected from mice and cattle were used in proliferation assays using either ^3^H-thymidine incorporation or PKH (Sigma-Aldrich) fluorescence intensity reduction as a read-out. For ^3^HT uptake experiments, 2.5 × 10^5^ cells were loaded per well in a 96-well round bottom plate (Thermo Scientific) in 200 μl of complete medium composed of RPMI 1640 + GlutaMAX (Invitrogen) supplemented with 50 μg/ml Gentamycin (Invitrogen), 50 μM β-mercaptoethanol (Sigma-Aldrich) and 10% fetal calf serum (Moregate). Each well was either stimulated with medium alone, 5 μg/ml nASP, 5 μg/ml pASP or 1 μg/ml ConA (Sigma-Aldrich), which served as positive control. Each condition was performed in triplicate. After 4 days of culture (cattle) or 5 days of culture (mice), cells were pulsed with 1 μCi ^3^HT (Perkin Elmer). After an additional 18 hours of culture, cells were harvested and analysed with a 1450 Microbeta β-scintillation counter (Perkin Elmer). Results are shown as stimulation index (SI), which is the ratio of the counts per minute of cells cultured with the vaccine antigen and the counts per minute of cells cultured with medium alone, or as counts per minute (CPM).

For the PKH experiments, cells were labelled with PKH26 (Sigma-Aldrich) according to the manufacturer’s instructions with the only difference that PKH26 was used at a dilution of 1/125. After labelling, a small fraction of the cells was used for flow cytometric analysis to determine the starting intensity of PKH. The rest of the cells were seeded at 2.5 × 10^5^ cells/200 μl RPMI complete medium (cattle) or at 5 × 10^5^ cells/200μl RPMI complete medium (mice) in 96-well round bottom plates, either stimulated with medium alone, 5 μg/ml nASP or 5 μg/ml pASP. After 5 days of culture, the cells were harvested, stained with monoclonal antibodies and analysed by flow cytometry. ModFit LT software (Verity Software House) was used to calculate the proliferation index for the different cell populations, based on the PKH data.

In order to measure cytokine production during the proliferation of bovine PBMCs, blood was collected 2 weeks after the second vaccination in study 2 and PBMCs were cultured in 24-well plates (BD Bioscience) at 1 × 10^6^ cells/ml in RPMI complete medium and re-stimulated *in vitro* with medium alone or 5 μg/ml of nASP for the nASP+QuilA, nASP+Al(OH)_3_ and PBS groups, or pASP for the pASP+QuilA group. After 5 days of culture, supernatants were harvested and used on an ELISA. IFNγ and IL4 capture monoclonal antibodies were coated in carbonate buffer (pH 9.6) on 96-well Maxisorp (Nunc) plates at a concentration of 2 μg/ml and 4 μg/ml, respectively. Plates were blocked with 2% BSA in PBS for one hour at room temperature. Afterwards, 50 μl of supernatant was administered to each well, and medium with a known concentration of either bovine IFNγ or IL4 was used as a standard. All conditions were performed in duplicate. Biotinylated monoclonal antibodies raised against IFNγ or IL4 (kindly provided by Prof. Jayne Hope, The Roslin Institute, UK) were added at a concentration of 2 μg/ml. Streptavidin-horseradish-peroxidase (Sigma-Aldrich) was used as a conjugate. O-phenylenediamine (Sigma-Aldrich) 0.1% in citrate buffer (pH 5.0) served as substrate. Optical density was measured at 492 nm. OD values were converted to concentrations using Deltasoft JV software (Deltasoft).

### Histology and cell counts

Abomasal tissue samples from the calves used in study 2 were collected and used for histochemical and immunostainings as previously described^18^. Globular leukocytes and mast cells were identified using Sirius Red staining (Polysciences inc.) and a Toluidine Blue staining (Sigma-Aldrich), respectively. The primary antibodies used for immunostainings were rabbit polyclonal anti-human CD3 (Dako), rabbit polyclonal anti-human CD20 (Thermo Scientific) and mouse IgG1 anti-human MAC387 (AbD Serotec). Secondary antibodies used were goat anti-rabbit IgG-biotin (Dako) or Goat anti-mouse IgG-biotin (Dako), diluted in PBS 2% BSA, according to the manufacturer’s instructions. Afterwards, sections were stained with the peroxidase-streptavidine complex (Dakocytomation A/S), diaminobenzidine tetrahydrochloride (DAB, Sigma–Aldrich) and H_2_O_2_ (Calbiochem) followed by counterstaining with haematoxylin. All sections were mounted in synthetic medium DPX prior to analysis.

B cells (CD20), T cells (CD3) and macrophages (MAC387) were quantified by taking 4 random pictures per tissue slide at 200x magnification and calculating the number of cells per mm^2^ tissue surface. Quantification of globular leucocytes and mast cells was carried out by counting the number of Sirius Red or Toluidine blue positive cells, respectively, present in 8 random fields of view per tissue slide at 400-x magnification. Cell counts were conducted for all animals of each group of vaccinated and infected animals. The results are expressed as cells/mm^2^.

### ELISA

For cattle, the systemic and abomasal IgG1, IgG2, and IgA levels against *O. ostertagi* nASP were determined by ELISA. nASP was coated in 96-well Maxisorp plates (Nunc) at a concentration of 0.5 μg/ml in carbonate buffer (pH 9.6). After 1 h blocking with 2% BSA-PBS at room temperature, either 100 μg mucus extract in PBS or 100 μl of a 1/200 serum dilution were administered in duplicate and incubated for an additional hour. After washing 3 times with 0.05% Tween 20-PBS, sheep anti-bovine IgG1, IgG2 and IgA (AbD Serotec) coupled to HRP were used as conjugates (dilution 1/500). O-phenylenediamine 0.1% in citrate buffer (pH 5.0) served as substrate. Optical density was measured at 492 nm.

The antibody responses in mice were measured by coating 96-well Maxisorp plates with nASP at a concentration of 5 μg/ml in carbonate buffer (pH 9.6). Following blocking with 2% BSA-PBS, 100 μl of a 1/200 serum dilution (were tested in duplicate. After washing 3 times with 0.05% Tween 20-PBS, rabbit anti-mouse IgG1, IgG2a (dilution 1/2000) and IgG2b (dilution 1/1000)(Sigma-Aldrich) and rat anti-mouse IgE (dilution 1/1000), all coupled to HRP, were used as conjugates and 2,2′-azinobis (3-ethylbenzthiazoline-6-sulfonic acid) (ABTS) in ABTS buffer (Roche) was used as substrate. Optical density was measured at 405 nm.

An inhibition ELISA assay was subsequently designed to evaluate the specificity of the antibodies raised in cattle. Serum samples from the nASP+QuilA vaccinated animals from study 1 were collected at one week after the final vaccination and used in an inhibition ELISA assay. Serum samples from all animals were pooled, diluted 1/200 and subsequently incubated for 1 h at room temperature with different concentrations of either nASP or pASP, ranging from 0 up to 500 pmol/ml, before being analysed by ELISA as described above.

### Statistical analysis

Statistical analyses were carried out using GraphPad Prism software. A non-parametric Kruskal-Wallis test was used to determine significant differences in parasitological parameters, cytokine production, antibody responses, cattle ^3^HT assays and cell frequencies between vaccinated and control groups. For the cattle ^3^HT assays, an additional ANCOVA (SPSS, IBM SPSS statistics version 23.0) was used to test correlation between weeks with no significant results. To determine significant differences in proliferation between the various cell subsets in the cattle abomasal MNCs and mice spleen MNCs, non-parametric Wilcoxon test was used. Finally, a non-parametric Friedman test was used to determine the statistical significance of the ^3^HT assays performed in mice where more than two groups were involved. A *P*-value of ≤0.05 was considered significant.

## Additional Information

**How to cite this article**: González-Hernández, A. *et al*. Host protective ASP-based vaccine against the parasitic nematode *Ostertagia ostertagi* triggers NK cell activation and mixed IgG1-IgG2 response. *Sci. Rep.* **6**, 29496; doi: 10.1038/srep29496 (2016).

## Supplementary Material

Supplementary Information
