# Supplementary material for: Host protective ASP-based vaccine against the parasitic nematode Ostertagia ostertagi triggers NK cell activation and mixed IgG1-IgG2 response
Source: Sci Rep. 2016 Jul 11;6:29496. doi: 10.1038/srep29496 (PMC4941725; doi:10.1038/srep29496)
Supplement: Supplementary Information [file srep29496-s1.pdf]

Host protective ASP-based vaccine against the parasitic nematode *Ostertagia ostertagi* triggers NK cell activation and mixed IgG1-IgG2 response

Ana González-Hernández, Stefanie Van Coppernolle, Jimmy Borloo, Frederik Van Meulder, Oonagh Paerewijck, Iris Peelaers, Georges Leclercq, Edwin Claerebout, Peter Geldhof

Legends to Supplemental Figures

Supplemental figure S1: Parasitological parameters

Faecal egg counts (FEC) were determined 3 times each week during the trickle infection until the time of necropsy, and expressed as number of eggs per gram faeces (EPG). For study 1 (A) and study 2 (B), mean cumulative FEC are shown for each animal in each group ± SEM. Statistically significant differences compared to control vaccinated animals are indicated with * ($p < 0.05$).

Supplemental figure S2. nASP-specific IgG1 and IgG2 antibodies observed in blood and mucosa after vaccination with nASP+QuilA, pASP+QuilA, and nASP+$Al(OH)_3$.

In cattle study 2, (A) serum and (B) abomasal samples were collected from all animals and used for the detection of nASP specific IgG1 and IgG2 type antibodies through ELISA. The graphs show the individual and mean (± SEM) OD's for each group. Statistically significant differences are indicated with * ($P < 0.05$).

Supplemental figure S3: Kinetics of the different immune cells during the vaccination period

In cattle study 2, PBMCs were isolated from each animal weekly during the vaccination period and the cells were stained with monoclonal antibodies to determine the frequencies of (A) αβ–T cells, (B) B cells, (C) NK cells and (D) γδ-T cells.

Supplemental figure S4: Phenotypical analysis of abomasal LN lymphocytes following vaccination and infection

Lymphocytes from abomasal LNs were isolated from the calves of study II and stained with monoclonal antibodies against CD3, TCRγδ and CD21 and CD335 and analyzed by flow cytometry. Individual and mean (± SEM) percentages of (A) αβ–T cells, (B) B cells, (C) NK cells and (D) γδ-T cells for each group are shown.

Supplemental figure S5: Immune cell counts in bovine abomasal tissue following vaccination and infection

Abomasal tissue samples were collected at the time of necropsy and either stained with antibodies to detect T cells ($CD3^{+}$), B cells ($CD20^{+}$) and macrophages ($MAC387^{+}$), or stained with Toluidine Blue or Sirius Red to detect mast cells and globular leukocytes respectively. The number of (A) macrophages, (B) B cells, (C) T cells, (D) globular leukocytes and (e) mast cells was determined per mm^2 in the mucosa. The graphs show the mean cell counts per mm^2 for the four animals within each group ± SEM.

Supplemental figure S6: Phenotypical analysis of intra epithelial and lamina propria lymphocytes isolated from the abomasum following vaccination and infection.

Abomasal intra-epithelial and lamina propria lymphocytes were isolated and stained with monoclonal antibodies against CD3, TCRγδ, CD21 and CD335 and analyzed by flow cytometry. Individual and mean (± SEM) percentages of (a) γδ-T cells, (b) αβ –T cells, (c) B cells and (d) NK cells are shown.

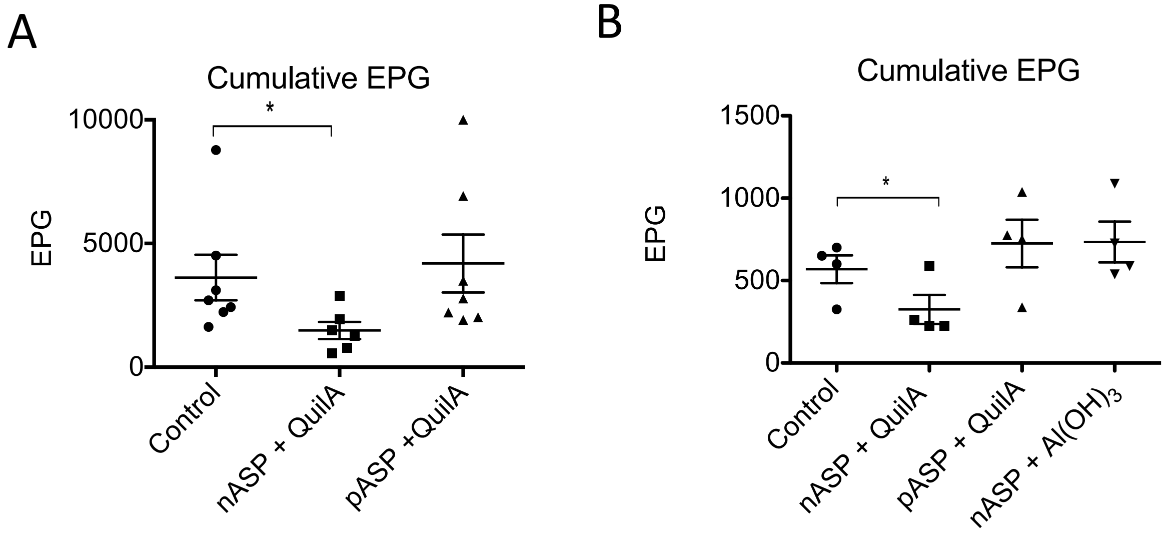

Supplemental Figure 1

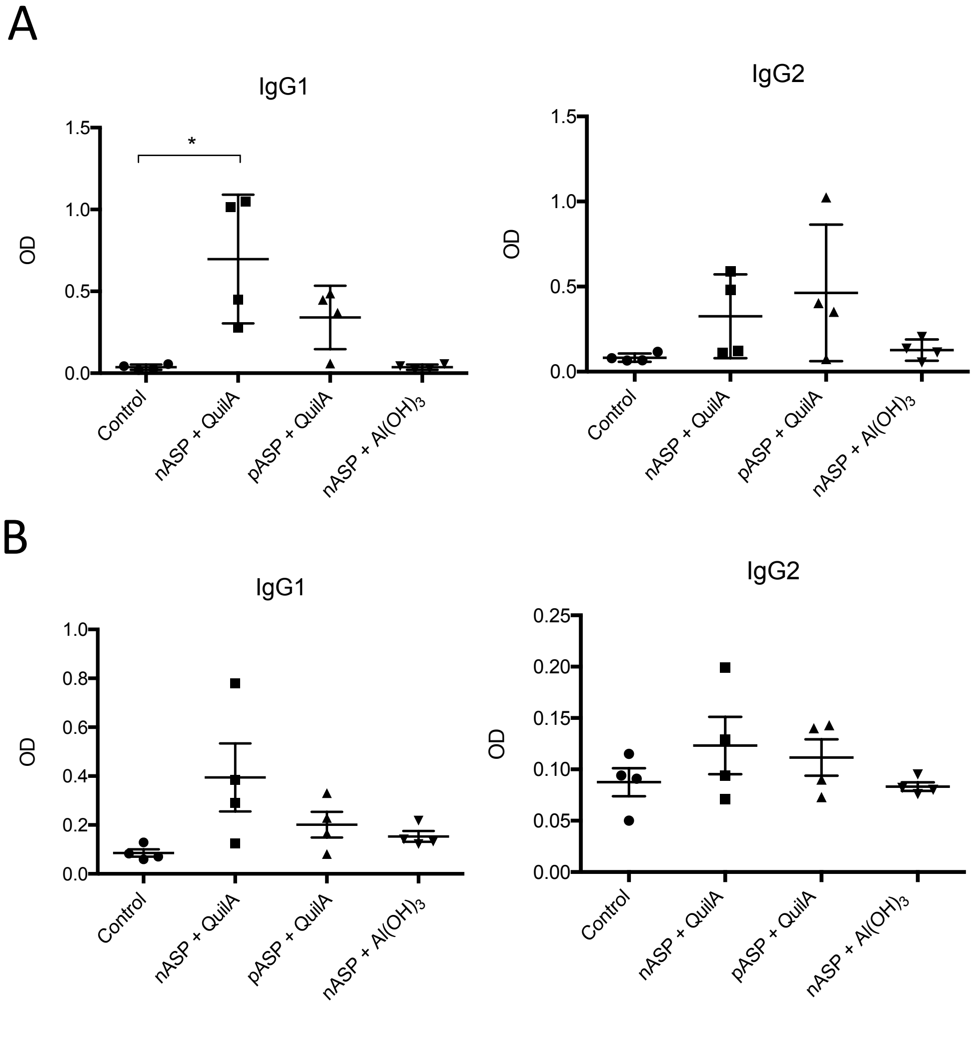

Supplemental Figure 2

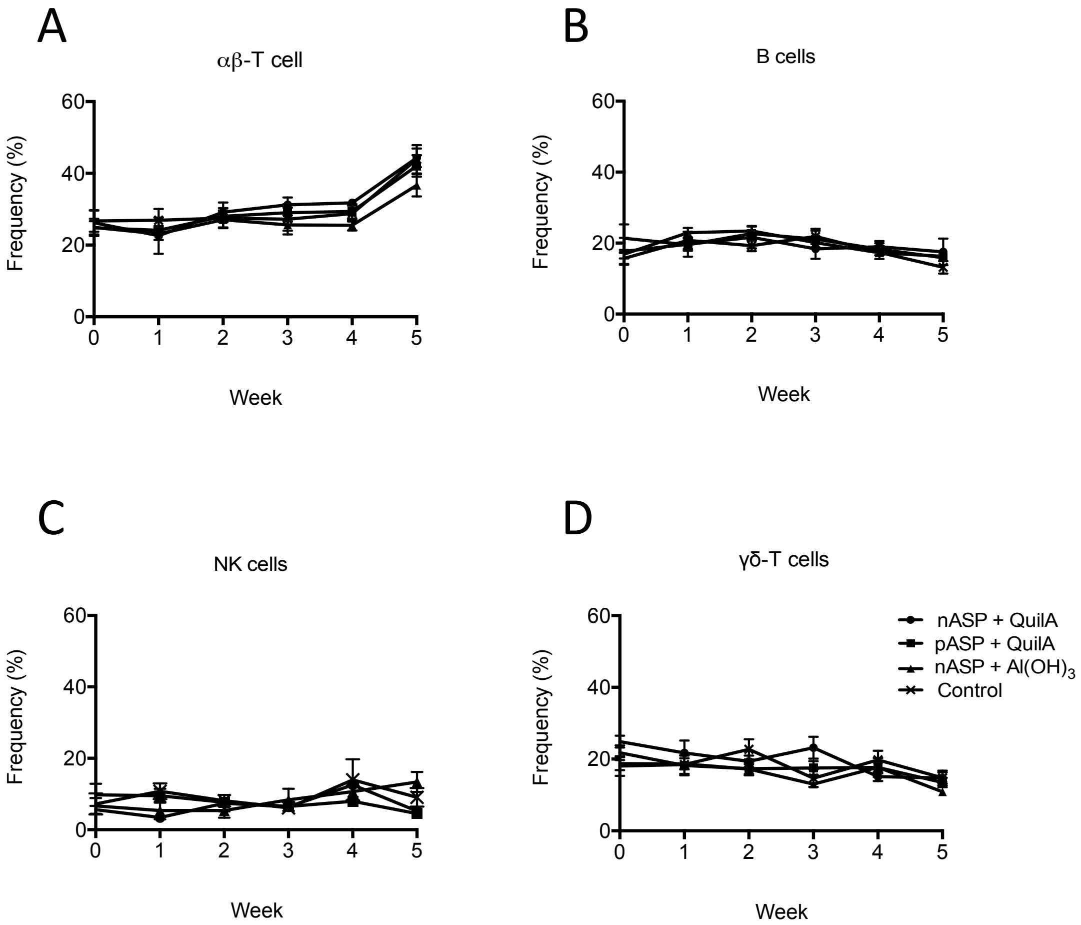

Supplemental Figure 3

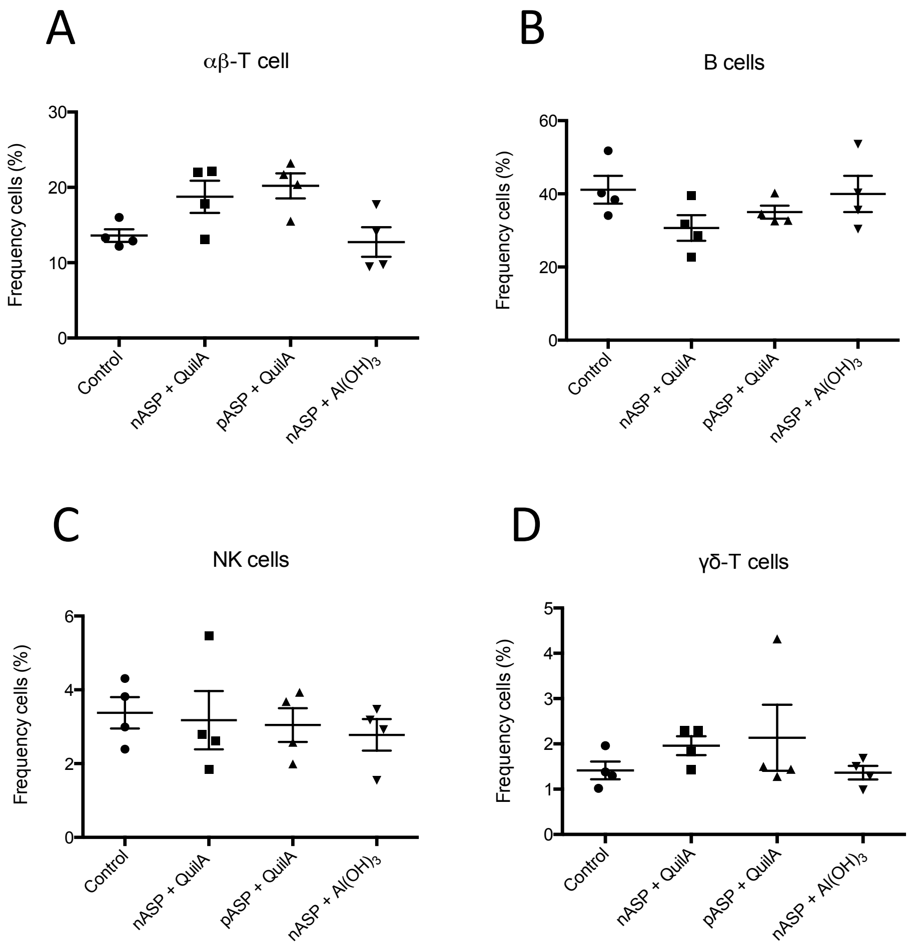

Supplemental Figure 4

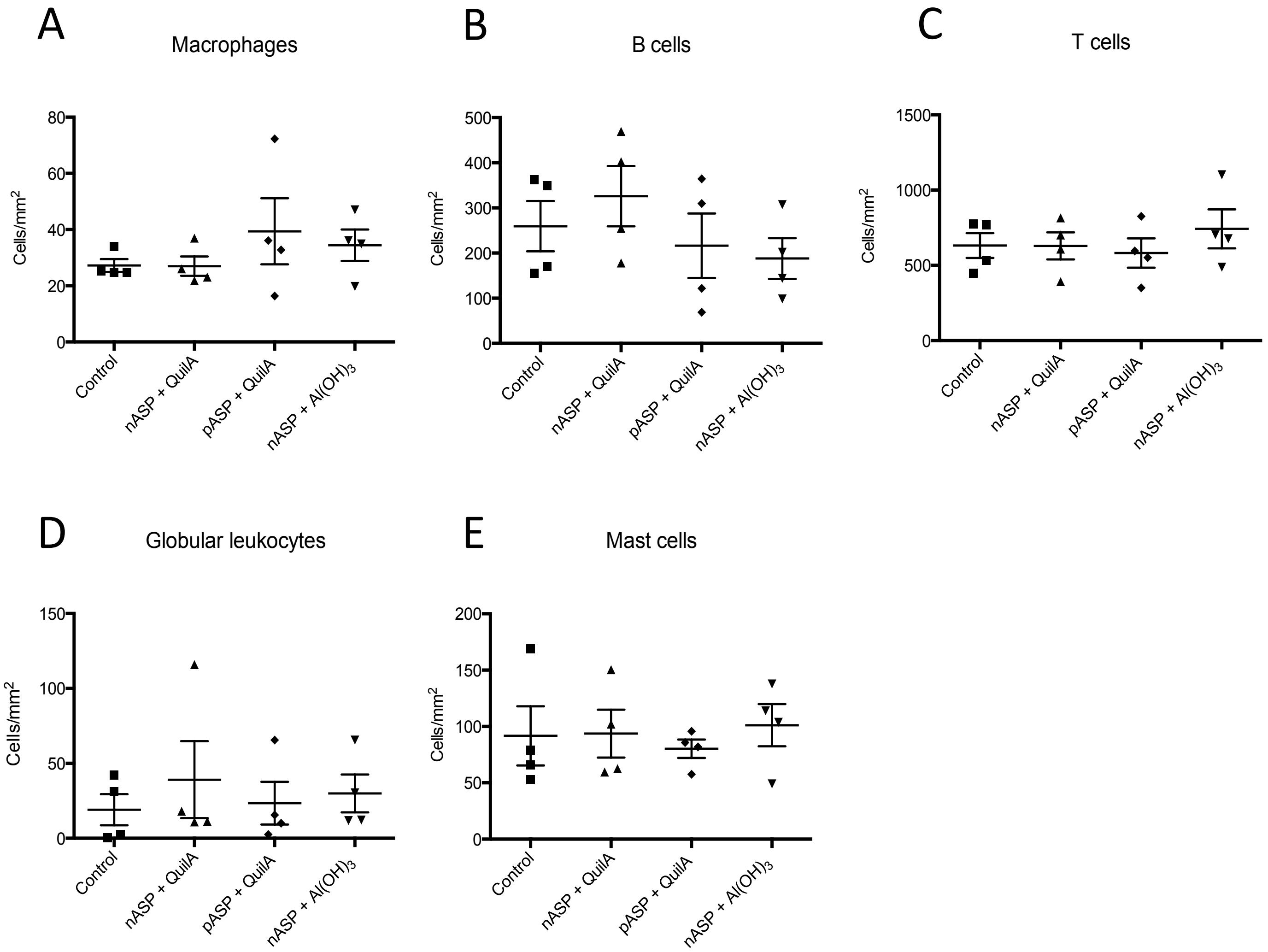

Supplemental Figure 5

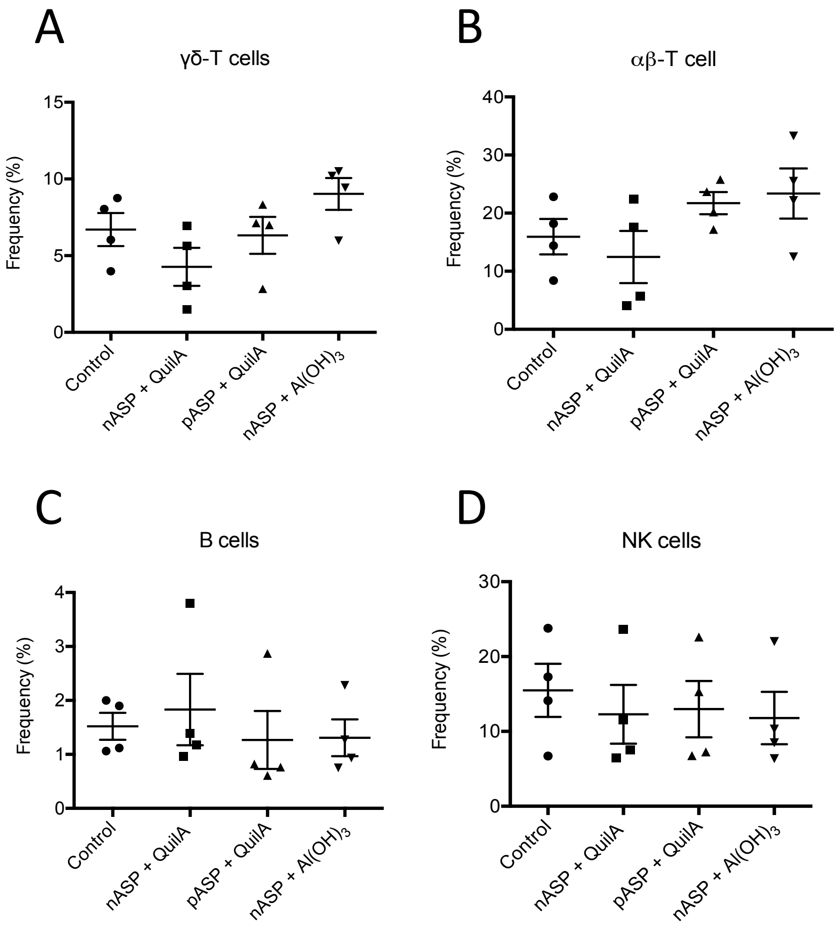

Supplemental Figure 6
